# Midbrain/pons area ratio and clinical features predict the prognosis of progressive Supranuclear palsy

**DOI:** 10.1186/s12883-020-01692-6

**Published:** 2020-03-30

**Authors:** Shi-Shuang Cui, Hua-Wei Ling, Juan-Juan Du, Yi-Qi Lin, Jing Pan, Hai-Yan Zhou, Gang Wang, Ying Wang, Qin Xiao, Jun Liu, Yu-Yan Tan, Sheng-Di Chen

**Affiliations:** 1 Department of Neurology & Institute of Neurology, Ruijin Hospital affiliated to Shanghai Jiao Tong University School of Medicine, Shanghai, China; 2 Department of Geriatrics, Ruijin Hospital Affiliated to Shanghai Jiao Tong University School of Medicine, Shanghai, China; 3 Department of Radiology, Ruijin Hospital Affiliated to Shanghai Jiao Tong University School of Medicine, Shanghai, China

**Keywords:** Progressive Supranuclear palsy, Survival analysis, M/P area ratio

## Abstract

**Background:**

Progressive supranuclear palsy (PSP) is a rare movement disorder with poor prognosis. This retrospective study aimed to characterize the natural history of PSP and to find predictors of shorter survival and faster decline of activity of daily living.

**Method:**

All patients recruited fulfilled the movement disorder society (MDS) clinical diagnostic criteria for PSP (MDS-PSP criteria) for probable and possible PSP with median 12 years. Data were obtained including age, sex, date of onset, age at onset (AAO), symptoms reported at first visit and follow-up, date of death and date of institutionalization. Magnetic resonance imaging was collected at the first visit. Endpoints were death and institutionalization. Kaplan-Meier method and Cox proportional hazard model were used to explore factors associated with early death and institutionalization.

**Results:**

Fifty-nine patients fulfilling MDS-PSP criteria were enrolled in our study. Nineteen patients (32.2%) had died and 31 patients (52.5%) were institutionalized by the end of the follow-up. Predictors associated with poorer survival were late-onset PSP and decreased M/P area ratio. Predictors associated with earlier institutionalization were older AAO and decreased M/P area ratio.

**Conclusion:**

Older AAO and decreased M/P area ratio were predictors for earlier dearth and institutionalization in PSP. The neuroimaging biomarker M/P area ratio was a predictor for prognosis in PSP.

## Background

Progressive supranuclear palsy (PSP) is a rare movement disorder with an estimated prevalence of 6.4 per 100,000, characterized by the accumulation of abnormally phosphorylated tau protein in the basal ganglia, frontal lobe and the brainstem [1]. The cardinal clinical features of PSP are early postural instability, supranuclear gaze palsy, akinesia, cognitive impairment and behavior changes [2].

The prognosis of PSP is poor with increased risks of falls, dysphagia, aspiration pneumonia and pressure ulcer, leading to institutionalization and short survival time [3]. The mean survival is only 3 to 4 years after the diagnosis and 5.3 to 13.0 years at the onset of the disease [1]. Previous studies indicated that male, older age at onset (AAO), early falls, dementia and clinical phenotypes were predictors of poor survival [1, 4–9].

Midbrain atrophy is commonly observed in PSP. A number of midbrain metrics including “hummingbird” sign, midbrain/pons (M/P) area ratio and magnetic resonance parkinsonism index (MRPI) have been proposed as potential neuroimaging biomarkers in PSP [10–13]. Neuroimaging biomarkers such as M/P area ratio are more objective and sensitive. It may provide more detailed information about progression of disease and therapeutic effects of anti-parkinsonism drugs, including levodopa. Thus, it is of value to examine the utility of neuroimaging biomarkers for the disease prognosis. A previous study found that MRPI predicted vertical supranuclear gaze palsy in patients with PSP [14]. But there is no study that has explored whether neuroimaging biomarkers can predict prognosis of PSP. We hypothesize that M/P area ratio indicates a poor prognosis in PSP.

Additionally, previous studies mainly focused on survival rather than activity of daily living which reflects the progression of the disability of living and is less influenced by factors such as nursing.

This study aimed to characterize the natural history of PSP and to find the predictors including neuroimaging biomarkers of shorter survival and faster decline of activity of daily living.

## Methods

### Participants

Patients were recruited at the Department of Neurology & Institute of Neurology, Rui Jin Hospital Affiliated to Shanghai Jiao Tong University School of Medicine from 2005 to 2016. We enrolled patients fulfilling the Neurological Diseases and Stroke-Society for PSP (NINDS-SPSP) clinical diagnostic criteria. We reconfirmed and corrected them with the movement disorder society (MDS) clinical diagnostic criteria for PSP (MDS-PSP criteria) for probable and possible PSP according to their disease history and information including neuroimaging [2]. The onset of the disease of all patients should be before Jan 2015 to ensure that we can explore for the presence of symptoms within 3 years of the disease onset. The study was approved by the medical ethics committee of Rui Jin Hospital affiliated to Shanghai Jiao Tong University School of Medicine. Participants and their caregivers were fully informed and signed written informed consent before the inclusion in the study.

### Data collection

Data was obtained from medical records or telephone interview including age, gender, date of onset, age at onset (AAO), interval from the onset to brain MRI, side of onset, symptoms reported at the first visit and the follow-up, date of death and the date of institutionalization. Information on the occurrence and time that began to fall, freezing of gait (FOG), bulbar palsy, apathy and urinary incontinence was collected. Patients with symptoms mentioned above within 3 years of the first symptom were defined as having an early onset (short latency) of those features [2, 8]. Patients were also assessed with Mini-Mental State Examination (MMSE) at the first visit.

Endpoints were death or institutionalization. Institutionalization was defined as Hohn-Yahr stage V. Disease duration to death was defined as the interval from disease onset to death or to the end of follow-up (June 2018). Disease duration to institutionalization was defined as interval from disease onset to reach endpoint (June 2018).

According to MDS-PSP criteria, patients were subdivided into PSP with Richardson’s syndrome (PSP-RS) and non-PSP-RS phenotype [2]. Non-PSP-RS phenotype includes PSP with predominant parkinsonism (PSP-P), PSP with predominant frontal presentation (PSP-F), PSP with progressive gait freezing (PSP-PGF), PSP with predominant ocular motor dysfunction (PSP-OM), PSP with predominant speech/language disorder (PSP-SL) [2].

### MRI acquisition and measurement

Non-contrast Brain MRI (GE, USA) was performed with 1.5-T MRI scanner included T1-weighted sagittal images, T1-weighted axial images and FLAIR axial images.

**Fig. 1:**
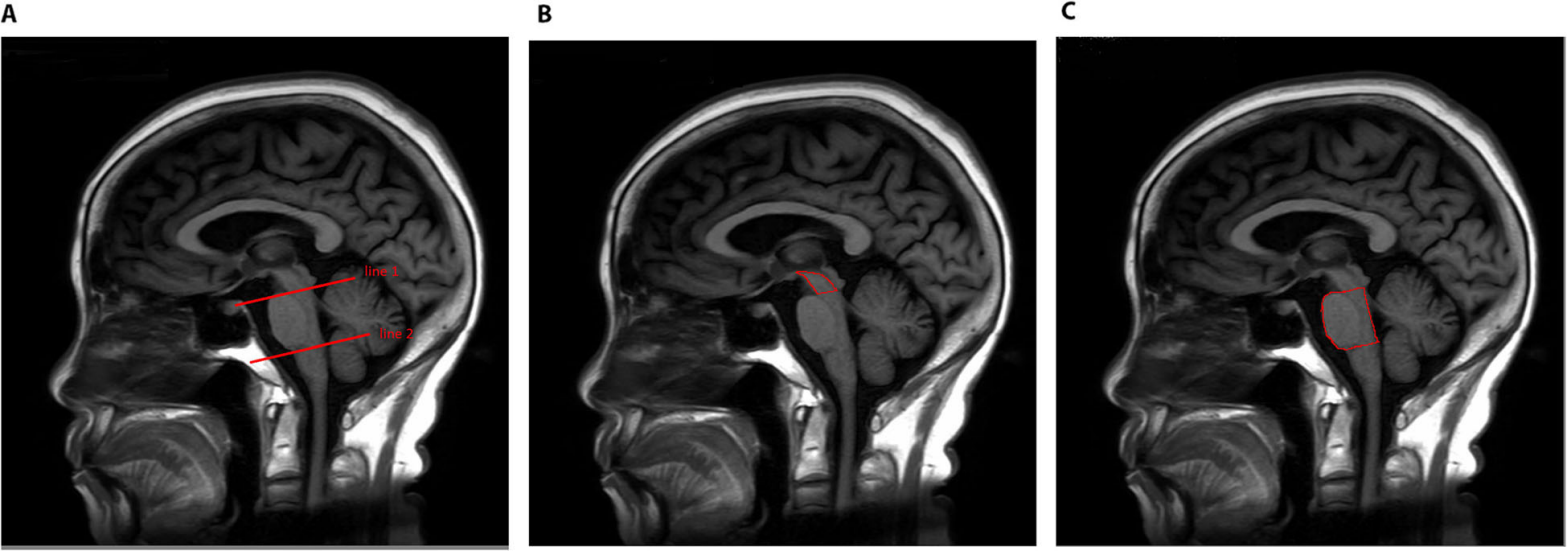
Sagittal T1-weighted MR images show midbrain area and pons area. **a** line 1 and line 2; **b** midbrain area; **c** pons area

The manual measurements were performed on midsagittal TI-weighted MRI with Mricro as shown in Fig. 1. The T1-weighted sagittal images were performed with echo time (TE) 9.09 ms, repetition time (TR) 2510.96 ms and voxel size 0.47*0.47*6mm^3^. The brainstem was divided into 3 sub-regions (midbrain, pons and medulla oblongata) on T1 midsagittal sections by drawing 2 cutting planes: the first line is between the superior pontine notch and inferior edge of the quadrigeminal plate (line1) and the second line is parallel to the first through the inferior pontine notch (line 2) (Fig. 1a) [10, 12, 13, 15].

The midbrain area was above line 1 (excluding the tectum) (Fig. 1b). The pons area was between the liquor- parenchyma-border of the pons, the anterior liquor-parenchyma-border of the fourth ventricle, line 1 and line 2 (Fig. 1c). M/P area ratio was calculated as the ratio of midbrain area to pons area.

Image analysis was performed by two independent raters. To assess the intrarater reliability, a second evaluation was made 1 week after the first evaluation by one of the two raters.

### Statistical analysis

Demographic and clinical variables were presented as frequency for categorical variables, or as the mean and standard deviation for continuous variables. Intraclass correlation coefficient was used to assess the intrarater and interrater reliability. Survival curves for each categorical variable were estimated by the Kaplan-Meier (K-M) method and differences in survival were measured by the log rank test. The risk of death or institutionalization was calculated using the Cox proportional hazard model in univariate analysis; variables associated with significance were entered in the multiple stepwise regression model. The Variance Inflation Factor (VIF) of variables included in multivariate analysis were all below 5. Hazard ratio (HR) and 95% confidence interval were calculated. Significance was tested at the 5% level.

## Results

Fifty-nine patients fulfilling MDS-PSP criteria were enrolled in our study. Clinical and demographic characteristics of patients are summarized in Table 1. 43 (72.9%) patients were classified as PSP-RS, while 16 (27.1%) were classified as non-PSP-RS including 11 patients with PSP-P, 2 with PSP-F and 3 with PSP-FOG. Nineteen patients (32.2%) had died and 31 patients (52.5%) were institutionalized by the end of the follow-up. The median survival time was 12.0 years and median time from onset of the disease to institutionalization was 9.0 years.

**Table 1:** Characteristics of patients with PSP

	PSP(***N*** = 59)	PSP-RS(***N*** = 43)	Non PSP-RS(***N*** = 16)
Gender (Male/Female)	34/25	26/17	8/8
Age (years)	64.7 ± 8.0	65.6 ± 7.6	62.4 ± 8.7
Age at onset	62.0 ± 2.3	63.5 ± 7.1	57.9 ± 9.6
The interval from the onset of symptoms to brain MRI (years)	3.8 ± 2.3	3.4 ± 2.2	4.7 ± 2.2
No. of death	19	16	3
Median survival (years)	12	12	15
No. of institutionalization	31	22	9
Median time from onset to institutionalization (years)	9	8	10
Early falls(%)	69.5	86.0	25.0
Early FOG(%)	30.5	30.2	31.3
Early bulbar paralysis (%)	66.1	79.1	31.3
Early apathy(%)	35.4	44.2	12.5
MMSE	23.0 ± 5.1	23.4 ± 3.9	21.9 ± 7.8
Early incontinence(%)	32.2	39.5	12.5
M/P area ratio(%)	21.3 ± 7.1	20.0 ± 6.7	24.3 ± 7.6

The mean M/P area ratio in MRI was 21.3% (Table 1). The correlation between intrarater for midbrain area was 0.991 and for pons area was 0,998. The correlation between interrater for midbrain area was 0.992 and for pons was 0.999.

Stratified by gender, phenotype and presence or absence of clinical features within 3 years, we found that patients with early falls, early bulbar palsy, early apathy and early incontinence showed shorter survival time (log-rank: *p* = 0.032, *p* = 0.013, *p* = 0.018, *p* = 0.001) compared to patients without this feature within 3 years. Patients with early bulbar palsy, early apathy, early incontinence showed shorter time from onset to institutionalization by K-M method (*p* = 0.016, *p* = 0.027, p = 0,003). In addition, a comparison between PSP-RS and PSP-P excluding PSP-F and PSP-FOG was also performed. Compared with PSP-RS, PSP-P had a longer survival in trend (log-rank: *p* = 0.067), but no significant difference was observed for institutionalization by K-M method (log-rank: *p* = 0.115).

In univariate analysis using the Cox proportional hazard model, early onset of falls, apathy, bulbar paralysis and urinary incontinence were associated with higher mortality risk (Table 2). Decreased M/P area ratio was associated with higher mortality risk after adjusting for age and duration from onset to visit. The selected multivariate regression model included gender, age at onset, early falls, early apathy, early bulbar palsy, early urinary incontinence and M/P area ratio adjusted by age. In this model, older age at onset (HR 2.087, P = 0.016) and decreased M/P area ratio  (HR 0.808, P = 0.005) were independently associated with higher mortality risk.

**Table 2:** Predictors for early death

	Univariate		Multivariate	
	HR (95%CI)	*P* value	HR (95%CI)	*P* value
Gender (M/F)	1.314 (0.492–3.511)	0.581		
Age at onset	0.999 (0.942–1.060)	0.970	2.087 (1.146–3.801)	0.016
phenotype (non-RS:RS)	0.417 (0.118–1.477)	0.417		
Early falls	3.554 (1.012–12.485)	0.048		
Early FOG	0.679 (0.238–1.932)	0.679		
Early bulbar paralysis	4.391 (1.212–15.905)	0.024		
Early apathy	3.040 (1.128–8.189)	0.028		
Early incontinence	4.958(1.704–14.430)	0.003		
MMSE	0.886 (0.755–1.040)	0.139		
M/P area ratio (%) ^a^	0.808 (0.697–0.937)	0.005	0.808 (0.697–0.937)	0.005

^a^ adjusted for age and interval from onset to brain MRI

In univariate analysis using the Cox proportional hazard model, early onset of apathy, bulbar palsy and urinary incontinence were associated with higher institutionalized risk (Table 3). Decreased M/P area ratio was associated with higher institutionalized risk after adjusting for age and duration from onset to visit. The selected multivariate regression model included gender, age at onset, early bulbar palsy, early apathy, early urinary incontinence and M/P area ratio adjusted by age. In this model, older age at onset (HR 1.476, P = 0.004) and decreased M/P area ratio (HR 0.925, P = 0.046) were independently associated with higher institutionalized risk.

**Table 3:** Predictors for early institutionalization

	Univariate		Multivariate	
	HR (95%CI)	*P* value	HR (95%CI)	*P* value
Gender (M/F)	1.716 (0.783–3.765)	0.178		
Age at onset	1.046 (0.993–1.101)	0.088	1.476 (1.133–1.924)	0.004
phenotype (non-RS:RS)	0.626 (0.274–1.430)	0.266		
Early falls	1.678 (0.760–3.705)	0.200		
Early FOG	0.649 (0.284–1.485)	0.306		
Early bulbar paralysis	2.485 (1.108–5.576)	0.027		
Early apathy	2.153 (1.021–4.536)	0.044		
Early incontinence	3.174 (1.369–7.360)	0.007		
MMSE	0.928 (0.812–1.061)	0.279		
M/P area ratio (%) ^a^	0.925 (0.856–0.999)	0.046	0.925 (0.856–0.999)	0.046

^a^ adjusted for age and duration from onset to visit

## Discussion

To the best of our knowledge, this paper is the first to consider the neuroimaging biomarker M/P area ratio as a predictor for prognosis and to explore the factors associated with the progression of disability of living. In this paper, we found that predictors associated with poorer survival and earlier institutionalization were older age at onset and decreased M/P area ratio.

In the survival analysis, decreased M/P area ratio was associated with earlier death and institutionalization. Paviour et al. found that M/P area ratio was smaller in PSP than Parkinson’s disease, multiple system atrophy or controls [16]. The phenomenon that midbrain atrophy progressed in PSP-RS and PSP-P was observed in previous researches [15, 17]. Previous studies found that brainstem atrophy rates, especially the change in the midbrain volume were correlated with disease progression measured by PSP Rating Scale [18–21]. Inability to move eyes downward early was observed to predict survival time and midbrain atrophy predicted the disease progression of supranuclear gaze palsy [14, 22]. These researches indicated that M/P area ratio may be related to progression of motor disability and death risk in PSP. Axial symptoms may influence the survival time and it is also associated with pathological change in the brainstem, especially in the midbrain, thus they are associated with a smaller midbrain volume [16]. Besides, the correlations were identified in previous studies between midbrain volume and motor disability and between PSPRS scores and [18F]AV-1451 uptake in the midbrain, indicating that pathological change in midbrain might predicted severer disease [16, 23]. Thus, it might be useful as a progression marker in clinical trial for PSP.

In accordance with most previous studies, older age at onset is associated with shorter survival [6–9, 24]. But other few studies have found negative or inverse association between AAO and survival time [22, 25]. Shorter survival and institutionalization for people were expected for the elderly, regardless of disease [9]. In our study, we also observed older AAO was a predictor for early institutionalization, which means patients with older AAO is associated with more rapid progression in motor disability. Consistent with our results, Golb et al. found that patients with older AAO has a higher PSP rating scale score and progressing more rapidly [24].

Few studies investigated the association between urinary incontinence and survival time, though it was one of the prevalent autonomic symptoms in PSP [25–27]. In our study, we found the association between incontinence and survival and institutionalization in univariate model, but the association became negative in multivariate model. Rare studies explored the association between apathy and prognosis in PSP despite that apathy is a common psychiatric symptom in PSP. In our study, we found that apathy was associated with death and institutionalization in earlier institutionalization in univariate model, but the association became negative in multivariate model. But the sample of our study was small, larger sample is needed to verify our results.

Inconsistent with previous studies found that RS phenotype was associated with a worse prognosis [4, 5, 8], there was no significant association found between phenotype and prognosis with K-M method or Cox proportional hazard model in our study. The reasons for the inconsistence are as followings. Previous studies mostly compared PSP-RS phenotype versus PSP-P phenotype while non-RS phenotype explored in our study included PSP-P, PSP-F and PSP-FOG. Published studies found that the median survival time of patients with PSP-F was similar with PSP-RS and its cumulative mortality after 5 years was mildly higher than PSP-RS [28–30]. The dementia and frontal symptoms, the most common features in PSP-F, were associated with earlier death [26, 31]. A recently published study found cognitive impairment in PSP, especially executive dysfunction, was associated with severity of PSP-related tau pathology in autopsy-confirmed PSP patients, which might explain the association with poorer prognosis [32]. After excluding PSP-F and PSP-FOG, we found that a trend for the association of PSP-P with a longer survival. The small number of our sample may also influence our results. Thus, a larger sample with various phenotype is needed for further exploration. Besides, the MDS-PSP criteria was used in this study, while previous studies using NINDS-SPSP. This difference of two diagnosis criteria may account for the negative results to some extent.

Furthermore, unlike in previously published articles, the criteria for diagnosis and classification in the study was MDS-PSP rather than NINDS-SPSP. Respondek et al. found that the sensitivity of NINDS-SPSP to detect non-RS phenotype was relatively unsatisfactory [29]. MDS-PSP is based on NINDS-SPSP added akinesia and cognitive dysfunction besides ocular motor dysfunction, postural instability as core symptoms, thus leading to the improved detection for non-RS phenotype [2].

The strength of our study are as follows: 1) the present study is the first one to investigate the association between neuroimaging and the prognosis in PSP; 2) in addition to explore the factors associated with early death, we also explored the factors associated with early institutionalization to find out what factors influence progression of disability; 3) some non-motor symptoms including apathy and urinary incontinence rarely mentioned in previous studies were included in our research to explore their role on survival and progression in PSP; 4) MDS-PSP criteria was used in our study to contain a wider spectrum of PSP.

Our study has several limitations. First, this was a retrospective study based on medical history and interview. The information of medical history may be less accurate for the biased memory of patients and incomplete medical history interviewed by doctor. However, the information was completed by movement disorder specialists and reconfirmed by a structured interview with the patient or their caregivers, making it more accurate. Secondly, the sample in our study was relatively small. However, PSP is a rare disease per se. Thirdly, the diagnosis was made by clinical criteria. However, the patients enrolled fulfilled the probable and possible PSP of PSP-MDS criteria which has relatively high specificity [2]. Besides, the mean interval between onset of symptoms and MRI was 3.8 years, which was a long time, since atypical clinical features and structural neuroimaging findings may not appear in the early disease course, interfering the diagnosis of PSP. Thus, an early neuroimaging marker are needed.

## Conclusion

Older AAO and decreased M/P area ratio were predictors for earlier death and earlier institutionalization in PSP. The neuroimaging biomarker M/P area ratio was a predictor for prognosis in PSP. Prospective studies with larger sample size will be needed further to confirm the predictors for prognosis.

## Data Availability

All data generated or analysed during this study are included in this published article.

## Abbreviations

AAO: Age at onset
FOG: Freezing of gait
HR: Hazard ratio
M/P: Midbrain/pons
MDS: The movement disorder society
MMSE: Mini-Mental State Examination
MRPI: Magnetic resonance parkinsonism index
NINDS-SPSP: The Neurological Diseases and Stroke-Society for PSP
PSP: Progressive Supranuclear Palsy
PSP-CBS: PSP with predominant corticobasal syndrome
PSP-F: PSP with predominant frontal presentation
PSP-OM: PSP with predominant ocular motor dysfunction
PSP-P: PSP with predominant parkinsonism
PSP-PGF: PSP with progressive gait freezing
PSP-RS: PSP with Richardson’s syndrome
PSP-SL: PSP with predominant speech/language disorder
VIF: Variance Inflation Factor
